# Methylenetetrahydrofolate reductase C677T polymorphism and the risks of polycystic ovary syndrome: an updated meta-analysis of 14 studies

**DOI:** 10.18632/oncotarget.18472

**Published:** 2017-06-14

**Authors:** Lihong Wang, Wenting Xu, Caihong Wang, Mengyu Tang, Yujia Zhou

**Affiliations:** ^1^ From the Zhangjiagang Hospital of Chinese Medicine, Nanjing University of Chinese Medicine, Nanjing, People's Republic of China; ^2^ Zhangjiagang, The First Clinical College, Nanjing University of Chinese Medicine, Nanjing, People's Republic of China

**Keywords:** PCOS, methylenetetrahydrofolate reductase, MTHFR, meta-analysis, polycystein

## Abstract

Some studies have reported an association between the Methylenetetrahydrofolate reductase (MTHFR) C667T genetic variant and risk of polycystic ovary syndrome (PCOS), although the results remain controversial. A systematic search was conducted on PubMed, Web of Science, EMBASE, Ovid, Chinese National Knowledge Databases and WanFang databases with relevant keywords. Fourteen studies of sixteen distinct populations involving 1478 PCOS cases and used to conduct a meta-analysis. The T allele was not significantly associated with increased risk of PCOS [OR: 1.08; 95% CI: 0.96–1.21]. In the stratified analysis by ethnicity, the T allele significantly increases risks for the Asian [OR = 1.31; 95% CI: 1.09–1.58] population. No significant associations were detected for the Middle Eastern population [OR = 1.26; 95% CI: 0.96–1.67] and the T allele was found to be protective in the Caucasian population [OR = 0.82; 95% CI: 0.68–0.99]. In conclusion, this meta-analysis suggests that MTHFR C667T variant can increase, decrease, or have no effect on the risks of PCOS depending on the ethnicity.

## INTRODUCTION

Polycystic ovary syndrome (PCOS) is the most widespread form of female infertility and affects an estimated 10–20% of women of reproductive age [1]. A 1990 National Institutes of Health (NIH) conference defined PCOS as hyperandrogenism and/or hyperandrogenemia (HA) with oligoanovulation, excluding other endocrine diseases [2]. In 2003, the Rotterdam consensus expanded the diagnostic criteria to include at least two of the following features 1) clinical hyperandrogenism 2) oligo-anovulation and 3) polycystic ovaries (PCO). Recently, an NIH expert panel suggested the broader Rotterdam criteria is more suitable for the diagnosis PCOS [1].

Although the etiology of PCOS remains largely unknown, it is believed to be a complex polygenic disorder heavily influenced by environment risk factors (for example, high-fat diet) [3, 4]. Previous evidence has suggested that the disturbed cycle and homocysteine-methionine cycle, two closely intertwined processes folate cycle in ovarian dysfunction. Deficiency or excess of folates inhibits ovulation in immature superovulated rats [5]. Folate deprived monkeys have degenerated Graffian follicles and an increase in atretic and cystic follicles [6]. Clinical evidence has also suggested that there are homocysteine levels in PCOS cases and homocysteine levels returned to normal levels following folic acid supplementation [7–9]. This makes Methylenetetrahydrofolate reductase (MTHFR) an essential gene to investigate in PCOS, as it has important roles in the processing of 5,10-methylenetetrahydrofolate to 5-methyltetrahydrofolate, a reaction required to convert homocysteine to methionine [10–12]. Some of its variants have show decreased efficiency of the folate/homocysteine pathway and have been linked to the susceptibility of neural tube defects, Alzheimer's disease, colon cancer, acute leukemia, and PCOS [13].

Experimental data have suggested that the functional substitution C677T (alanine substituted by valine, rs1801133) in the MTHFR gene reduces the activity of the folate pathway by 50% [10, 14, 15]. However, clinical studies thus far have found conflicting results regarding the association of MTHFR and PCOS [16–29]. To clarify the in-conflict findings reported so far as well as heterogeneity and publication bias that exists between studies, we have conducted a meta-analysis of genetic association studies of the MTHFR C677T polymorphism to assess its effect on the risk of PCOS.

## RESULTS

### Study characteristics

**Figure 1:**
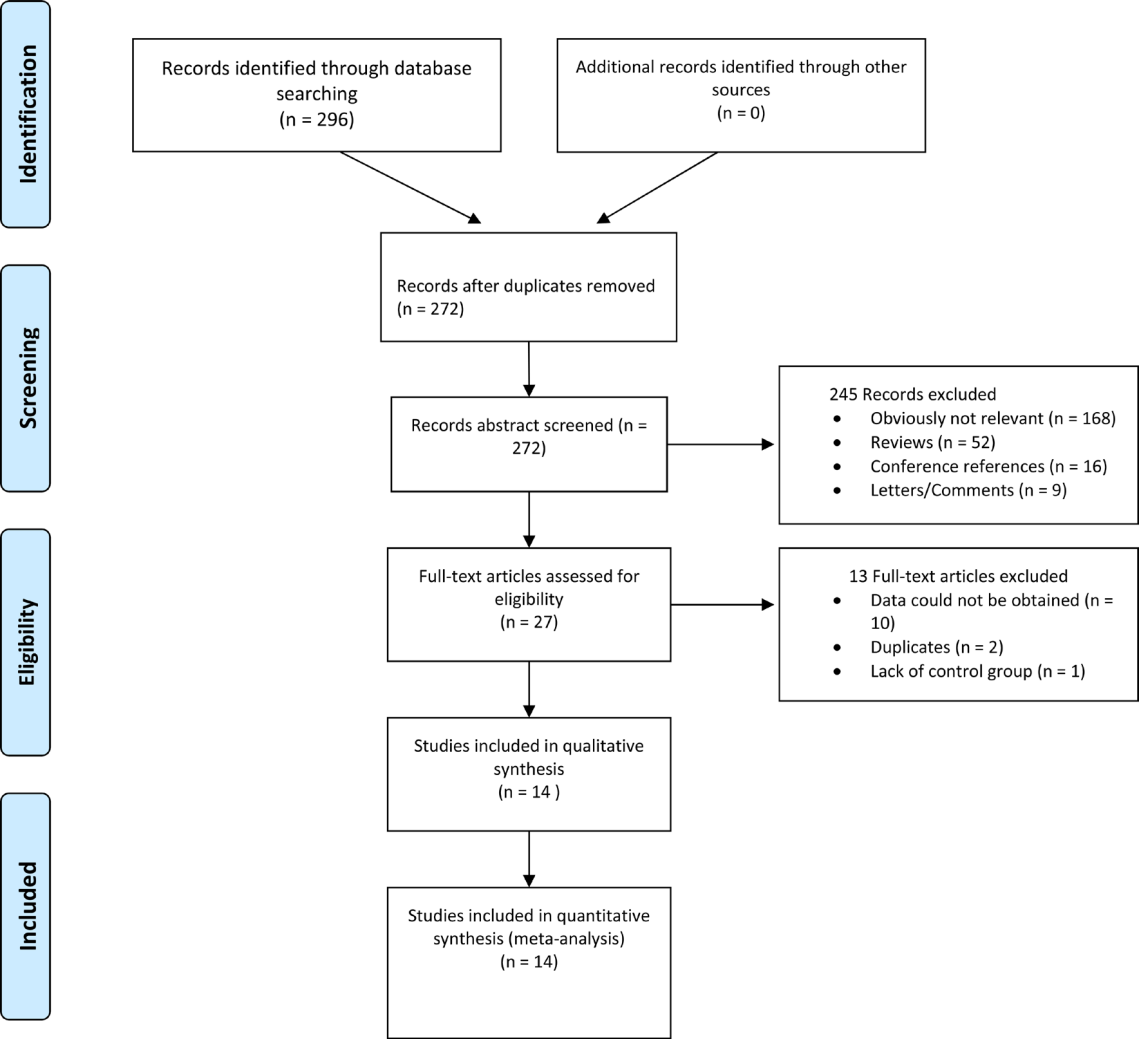
Flowchart of study selection

The search yielded a combined 272 references. Study selection process was shown in Figure 1. The final meta-analysis included a total of 14 articles of 16 data sets [16–29]. The 16 data sets included 1628 controls and 1478 PCOS cases. The detailed characteristics of included studies are shown in Table 1. Of the PCOS cases, 439 were Asian, 517 were Caucasian, 522 were Middle Eastern.

**Table 1:** Characteristics of included studies

Study	Year of publication	Diagnostic Criteria	Genotyping Method	Control	Average age (Case/Control)	Average BMI	Number of (case/Control)	Genotype Distribution (CC/CT/TT)		P(HWE)	Newcastle-Ottawa
								Case	Control		
Glueck [16]	1999	NIH 1990	RFLP	Population control	NA/37	NA/NA	41/234	14/23/4	119/89/26	0.2167	6
Sills [17]	2001	NIH 1990	RFLP	Healthy Control	29.6/32	33.5/27.5	36/18	25/9/2	8/9/1	0.3519	5
Tsanadis [18]	2002	NIH 1990	RFLP	Healthy Control	22.57/22.40	24.20/24.06	30/35	12/14/4	20/19/6	0.9791	6
Orio [19]	2003	Rotterdam Concensus	RFLP	Healthy Control	22.5/21.9	24.1/23.8	70/70	16/41/13	17/38/15	0.1464	7
Palep-Singh (Asian) [20]	2007	Rotterdam Concensus	RFLP	NA	30.3/33.4	28.6/25	21/9.0	14/7/0	9/0/0	0.3594	6
Palep-Singh (Caucassian) [20]	2007	Rotterdam Concensus	RFLP	NA	30.6/34.3	31.2/27.7	25/16	11/12/2	10/5/1	0.6068	6
Choi [21]	2009	Rotterdam Concensus	RFLP	Healthy Control	NA/NA	22.96/20.95	227/115	67/125/35	33/67/15	0.0625	5
Karadeniz [22]	2010	Rotterdam Concensus	TaqMan	Healthy Control	24.27/26.41	24.41/23.35	86/70	15/65/6	35/28/7	0	6
Idali [23]	2012	Rotterdam Concensus	RFLP	Healthy Control	29.6/NA	27.7/NA	71/100	36/31/4	66/25/92	0.4193	7
Jain [24]	2012	Rotterdam Concensus	RFLP	Healthy Control	24.19/24.31	28.13/23.18	92/95	76/16/0	82/13/0	0.361	6
Jiang [25]	2015	Rotterdam Concensus	TaqMan	Healthy Control	NA	NA	90/122	13/37/40	13/56/53	0.3601	4
Ozegowska [26]	2015	Rotterdam Concensus	TaqMan	Healthy Control	27.2/28.1	23.7/21.7	164/108	87/52/29	53/37/9	0.0001	6
Szafarowska [27]	2016	Rotterdam Concensus	Rotterdam	non-PCOS	33/36	NA/NA	76/56	33/39/4	19/30/7	0.0794	6
Carlus (Indu-European Population) [28]	2016	Rotterdam Concensus	Sequencing	Healthy Control	NA/NA	NA/NA	93/100	77/16/0	83/16/1	0.3641	6
Geng [29]	2016	Rotterdam Concensus	RFLP	Hospital Control	26.50/26.84	24.35/20.65	175/236	51/79/54	102/96/38	0.0557	5
Carlus (Dravidian Population) [28]	2016	Rotterdam Concensus	Sequencing	Healthy Control	NA/NA	NA/NA	168/156	132/33/3	126/29/5	0.5796	6

### Meta-analysis results

**Figure 2:**
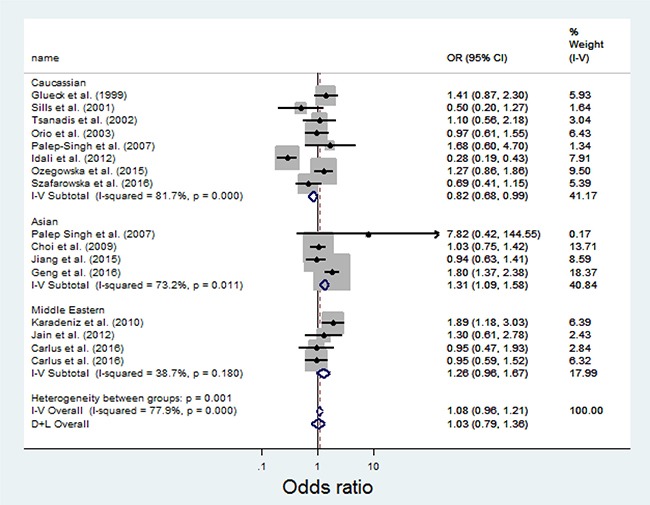
Meta-analysis of the association between MTHFR C667T and PCOS

Overall, there was no evidence of an association between the T allele variant and increased risks of PCOS when all data sets were pooled together. The per-allele OR of Pro using the random effects models was 1.08 [95% CI: 0.96–1.21; P(Z) = 0.000; P(Q) = 0.001; Figure 2]. The main results of the meta-analysis were listed in Table 2.

**Table 2:** Meta analysis of the MTHFR C667T polymorphism and the risks of PCOS

Total/Subgroup	Number of data sets	Number of cases/controls	C Allele		
			OR (95% CI)	P(Z)	P(Q)
Total	16	2980/7054	1.08 (0.96–1.21)	0.229	0.000
Caucasian	8	517/482	0.82 (0.68–0.99)	0.040	0.000
Asian	4	522/721	1.31 (1.09–1.58)	0.004	0.011
MiddleEastern	4	439/425	1.26 (0.96–1.67)	0.101	0.180

In the stratified analysis by ethnicity, the T allele significantly increases risks for the Asian [OR = 1.31; 95% CI: 1.09–1.58] population (See Figure 2). No significant associations were detected for the Middle Eastern [OR = 1.26; 95% CI: 0.96–1.67]. The T allele was found to be protective in the Caucasian population [OR = 0.82; 95% CI: 0.68–0.99].

In the dominant model, The CT + TT genotypes are significantly associated with increased risks of PCOS in the Asian [OR = 1.37; 95% CI: 1.02–1.83] and Middle Eastern [OR = 1.48; 95% CI: 1.06–2.07] populations. No significant associations were detected for the Middle Eastern [OR = 0.94; 95% CI: 0.73–1.21].

### Sensitivity analysis

**Figure 3:**
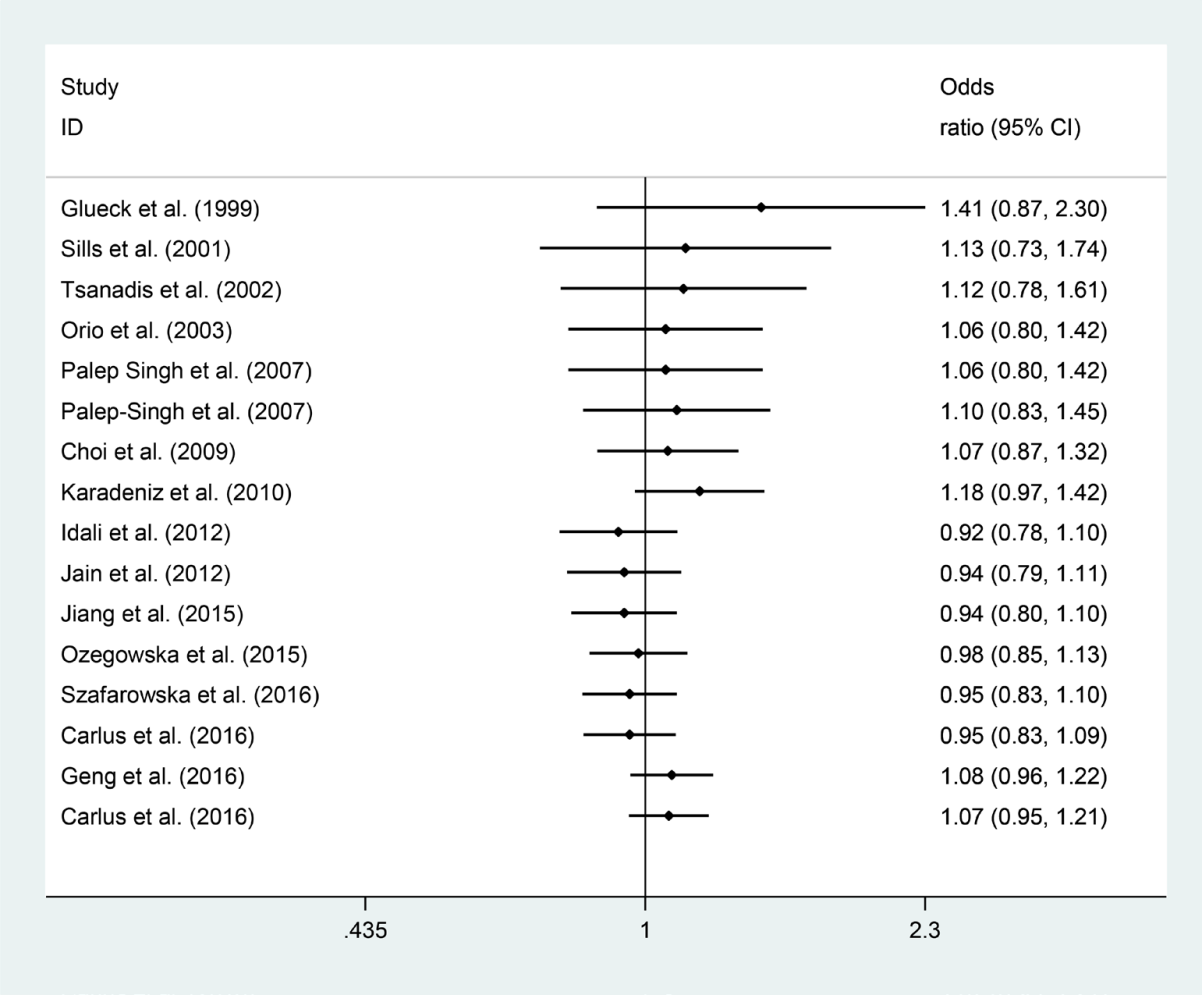
Cumulative meta-analysis of the studies included

**Figure 4:**
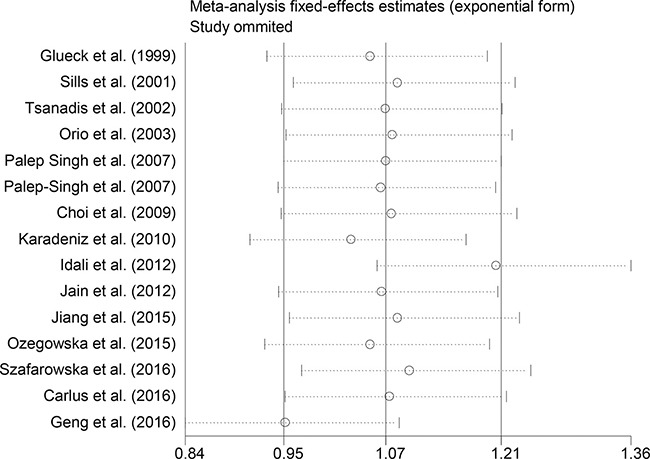
Sensitivity analysis excluding each included study one at a time

Sensitivity analyses using single-study omission demonstrated that this meta-analysis was stable (Figure 3). Statistical significance of the summary ORs was not modified (data not shown). A cumulative meta-analysis (Figure 4) also show that the results of this study are stable.

### Publication bias

**Figure 5:**
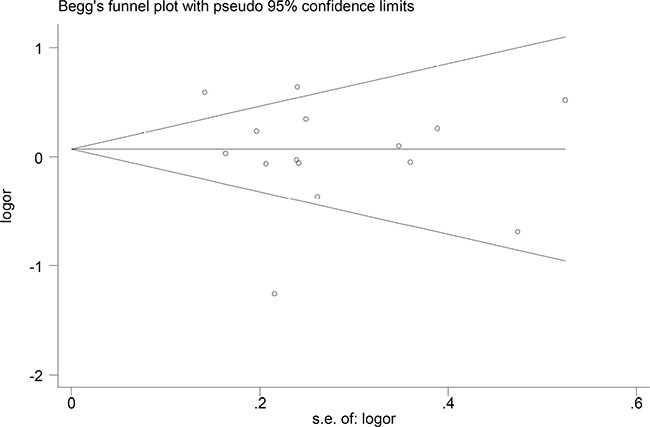
Begg's funnel plot of the included studies

**Figure 6:**
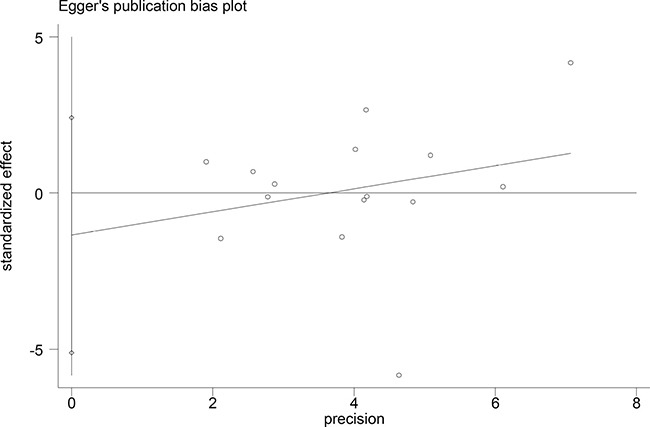
Egger's plot of the association of the included studies

Begger's and Eggar's funnel plots were constructed using the standard error and compared against the OR of each study (Figures 5 and 6). The plots do not suggest the existence of publication bias towards positive findings in smaller studies. Thus, this indicates that this meta-analysis is statistically robust.

## DISCUSSION

MTHFR is an enzyme that catalyzes the production of 5,10-methylenetetrahydrofolate to 5-methyltetrahydrofolate that is critical to the remethylation to methionine. Recently, the C677T variant has been linked with increased risks of PCOS, although to inconsistent results [16–29]. Due to small sample sizes, data generated by the above studies lacked the power of independent representation.

Previous meta-analyses analyzing the relationship between MTHFR C667T polymorphism and PCOS have yielded conflicting results. Bagos analyzed six data sets published before 2009 and found no correlation between C667T and the risk of PCOS [30]. However, the credibility of the study should be re-examined as the some of the values of homozygous rare and homozygous common genotypes have been interchanged. Fu and his colleagues performed a meta-analysis in 2014 analyzing 638 cases and 759 controls and reported that C667T as a risk factor for PCOS [31]. More recently, in 2015 Carlus and colleagues studied 960 cases and 1028 controls and found no correlation between C667T and the risks of PCOS [28].

Our up-to-date meta-analysis summarizes the evidence to date regarding the association between the MTHFR C677T variant and PCOS using a total of 1628 controls and 1478 PCOS cases, the largest to date by far. Overall, our results suggest that C677T is not associated with the risk of PCOS.

In our stratified analysis by ethnicity, a strong association was observed for the Asian population [OR: 1.31, 95% CI: 1.09–1.58] population but not for the Middle Eastern [OR = 1.26, 95% CI = 0.96–1.67] populations. Interestingly, risk T allele has protective effects for the Caucasian population [OR = 0.82, 95% CI = 0.68–0.99]. These results indicate that the association of the MTHFR C677T polymorphism and PCOS pathogenesis has deep genetic and possibly environmental background factors. Other factors such as differences in selection bias and matching criteria could also play a role in the difference between ethnic groups. It should also be noted that many studies included have very small study size. This suggests the possibility that the observed differences may be due to chance. Thus, additional studies are required to increase the statistical power and validate the racial difference of the MTHFR C677T polymorphism and PCOS risk.

The preferential publication of studies with positive results is a significant source of bias in many meta-analyses. However, the included studies in our meta-analysis also consist of studies with negative conclusions. Furthermore, our funnel plots do not show asymmetry, suggesting the lack of publication bias in our meta-analysis.

Several limitations should be noted in interpreting our results. We were not able to adjust for potential confounding effects conferred by gender, environmental factors, and lifestyle due to the lack of data. Our results were based on unadjusted estimates - a more precise analysis could be conducted if all raw data were available. The lack of individual health and metabolic data, such as drug history, diet, and body weight, also forbid us from performing a more sensitive analysis.

In conclusion, the pooled results of our meta-analysis indicate that C677T is not associated with the risks of PCOS. However, in the Middle Eastern populations, the T allele is strongly associated with the risks for PCOS while protective in Caucasian populations. Larger association studies with strict selection criteria are required to validate this result.

## MATERIALS AND METHODS

### Search strategy and inclusion criteria

We searched the literature hosted on PubMed, Web of Science, EMBASE, Ovid, Chinese National Knowledge Databases and WanFang with keywords related to disease (e.g. “Polycystic ovary syndrome,” “PCOS”) and the gene of interest (e.g. “Methylenetetrahydrofolate reductase”, “MTHFR”). Genetic association studies published before Dec 2016 were retrieved. The last search was performed on the Dec 28^th^, 2016. For each study, we checked their references to identify other relevant publications. No earlier publication date limit was applied. We did not define a minimum number of patients as a criterion for a study's inclusion in this meta-analysis. The search was conducted without any restrictions on the language used and focused on human studies.

All retrieved study were screened, and all eligible studies included needed to satisfy each point of the following criteria: 1) original papers containing independent trials, 2) case-control or cohort study, 3) confirmation of PCOS according to either the NIH or Rotterdam diagnostic criteria, and 4) genotype distribution information or odds ratio (OR) with its 95% confidence interval (CI) and *P* value. The major reasons for exclusion of studies were overlapping data, review articles, case-only studies, and insufficient data for analyses.

### Data extraction

Data extraction was performed independently by two reviewers using a standard extraction form. All data were checked for internal consistency and disagreements were resolved through thorough discussion between all authors. If there doubts about the result of studies, the corresponding author of the study was calculated. For each study, the following were extracted from each article: first author's name, publication year, diagnostic criterion, definition and numbers of cases and controls, frequency of genotypes, genotyping method, source of controls, Hardy–Weinberg equilibrium (HWE), age, body-mass index (BMI), and ethnicity. Studies with different ethnic groups within the same study were considered as individual studies for our analyses.

### Statistical analysis

The association strength between MTHFR C677T polymorphism and PCOS was assessed by calculating OR with 95% CI. The chi-square (χ^2^) test was used to evaluate whether there is a significant deviation from HWE among the control subjects of the study. The per-allele OR of risk allele T was compared between cases and controls in each study. The ORs were pooled using both the random-effects model (the DerSimonian and Laird method) and the fixed effects model (the Mantel-Haenszel method) as previously described [32, 33]. The Woolf's method was used to calculate 95% CI [34]. The results of the random effects model were reported in this article because it takes into consideration the variation between studies. A prespecified stratified analysis was conducted to explain the heterogeneity between each study and to investigate the relationship present in a subgroup. Stratified analysis was performed for ethnicity (Caucasian, Asian, and Middle Eastern).

Heterogeneity across individual studies was examined using Cochran's χ^2^ *Q* test [35]. *Q* test was also performed to detect the heterogeneity within each subgroup. Publication bias was assessed using linear regression to measure funnel plot asymmetry on the natural logarithm of OR using Egger's method [36]. All statistical analysis was carried out with Stata Version 13.0 (Stata Corporation, College Station, Texas, USA. All *P* values were for two-sided analysis. Type I error rate was set at 0.05.

## Abbreviations

BMI: body mass index
CI: confidence Interval
HWE: Hardy–Weinberg equilibrium
MTHFR: methyle netetrahydrofolate reductase
PCOS: polycystic ovary syndrome
OR: odds ratio
